# New World feline APOBEC3 potently controls inter-genus lentiviral transmission

**DOI:** 10.1186/s12977-018-0414-5

**Published:** 2018-04-10

**Authors:** Yoriyuki Konno, Shumpei Nagaoka, Izumi Kimura, Keisuke Yamamoto, Yumiko Kagawa, Ryuichi Kumata, Hirofumi Aso, Mahoko Takahashi Ueda, So Nakagawa, Tomoko Kobayashi, Yoshio Koyanagi, Kei Sato

**Affiliations:** 1 Laboratory of Systems Virology, Institute for Frontier Life and Medical Sciences, Kyoto University, Kyoto, Japan; 2 Graduate School of Biostudies, Kyoto University, Kyoto, Japan; 3 Graduate School of Pharmaceutical Sciences, Kyoto University, Kyoto, Japan; 4 Graduate School of Medicine, Kyoto University, Kyoto, Japan; 5 Faculty of Medicine, Kyoto University, Kyoto, Japan; 6 Faculty of Science, Kyoto University, Kyoto, Japan; 7 Faculty of Pharmaceutical Sciences, Kyoto University, Kyoto, Japan; 8 Micro/Nano Technology Center, Tokai University, Kanagawa, Japan; 9 Department of Molecular Life Science, Tokai University School of Medicine, Tokai University, Kanagawa, Japan; 10 Department of Animal Science, Faculty of Agriculture, Tokyo University of Agriculture, Kanagawa, Japan; 11 CREST, Japan Science and Technology Agency, Saitama, Japan; 12 Division of Systems Virology, Department of Infectious Disease Control, International Research Center for Infectious Diseases, Institute of Medical Science, The University of Tokyo, 4-6-1 Shirokanedai, Minato-ku, Tokyo, 1088639 Japan

**Keywords:** Lentivirus, FIV, APOBEC3, Vif, Evolutionary arms race, Puma, Bobcat, PLV, New World

## Abstract

**Background:**

The apolipoprotein B mRNA-editing enzyme catalytic polypeptide-like 3 (APOBEC3; A3) gene family appears only in mammalian genomes. Some A3 proteins can be incorporated into progeny virions and inhibit lentiviral replication. In turn, the lentiviral viral infectivity factor (Vif) counteracts the A3-mediated antiviral effect by degrading A3 proteins. Recent investigations have suggested that lentiviral *vif* genes evolved to combat mammalian APOBEC3 proteins, and have further proposed that the Vif-A3 interaction may help determine the co-evolutionary history of cross-species lentiviral transmission in mammals.

**Results:**

Here we address the co-evolutionary relationship between two New World felids, the puma (*Puma concolor*) and the bobcat (*Lynx rufus*), and their lentiviruses, which are designated puma lentiviruses (PLVs). We demonstrate that PLV-A Vif counteracts the antiviral action of APOBEC3Z3 (A3Z3) of both puma and bobcat, whereas PLV-B Vif counteracts only puma A3Z3. The species specificity of PLV-B Vif is irrespective of the phylogenic relationships of feline species in the genera *Puma, Lynx* and *Acinonyx*. We reveal that the amino acid at position 178 in the puma and bobcat A3Z3 is exposed on the protein surface and determines the sensitivity to PLV-B Vif-mediated degradation. Moreover, although both the puma and bobcat *A3Z3* genes are polymorphic, their sensitivity/resistance to PLV Vif-mediated degradation is conserved.

**Conclusions:**

To the best of our knowledge, this is the first study suggesting that the host A3 protein potently controls inter-genus lentiviral transmission. Our findings provide the first evidence suggesting that the co-evolutionary arms race between lentiviruses and mammals has occurred in the New World.

**Electronic supplementary material:**

The online version of this article (10.1186/s12977-018-0414-5) contains supplementary material, which is available to authorized users.

## Background

The apolipoprotein B mRNA editing enzyme catalytic polypeptide-like 3 (APOBEC3; A3) proteins are cellular cytidine deaminases that are specifically encoded in mammals but not in other vertebrates [1, 2]. Mammalian A3 proteins, particularly primate A3 proteins, are considered cellular intrinsic immune factors that potently restrict lentiviral replication. To exhibit antiviral activity, some A3 proteins are incorporated into the released progeny virions and enzymatically insert guanine-to-adenine hypermutations into the viral genome, thereby halting viral replication. In turn, the lentiviral protein, viral infectivity factor (Vif), antagonizes the A3-mediated antiviral action by degrading A3 proteins via the ubiquitin/proteasome-dependent pathway (reviewed in [3]).

Elucidating co-evolutionary relationships between hosts and their viruses is an intriguing topic in virology and is crucial to understanding how viruses influence their hosts’ evolution and vice versa. Cell-based virological experiments are essential for a better understanding of the evolutionary conflict between mammals and their viruses, including lentiviruses. For instance, since the interaction between host A3 and lentiviral Vif is species specific, various investigations focusing on the functional relationship between A3 and Vif have recently been conducted in combination with molecular phylogenetic approaches (reviewed in [4]). This strategy stems from the “Red Queen hypothesis [5]”, which proposes that host and viral proteins have competed with one another for survival over time [6–8]. However, since most previous observations are based the Old World evolutionary events [4], whether the evolutionary arms race between mammals and lentiviruses has occurred in the New World is unclear.

Feline immunodeficiency virus (FIV) is a lentivirus that was first isolated in 1987 from domestic cats (*Felis catus*) with chronic AIDS-like disorders [9]. Domestic cats encode multiple *A3* genes, and the feline A3 protein (designated A3Z3) potently impairs FIV replication by incorporating into nascent virions [10–15]. In response, FIV Vif antagonizes the antiviral activity of feline A3Z3 by degrading this protein [11–15]. To elucidate the evolutionary relationship between FIV and felids, we have recently reported that the domestic cat *A3Z3* is polymorphic and that a haplotype of the domestic cat A3Z3 is resistant to Vif-mediated degradation by FIVfca, which is the FIV that infects domestic cats [12]. Our findings suggest that the domestic cat *A3Z3* is under positive selection due to evolutionary selective pressure caused by FIVfca or related ancestral viruses [12, 14].

**Fig. 1:**
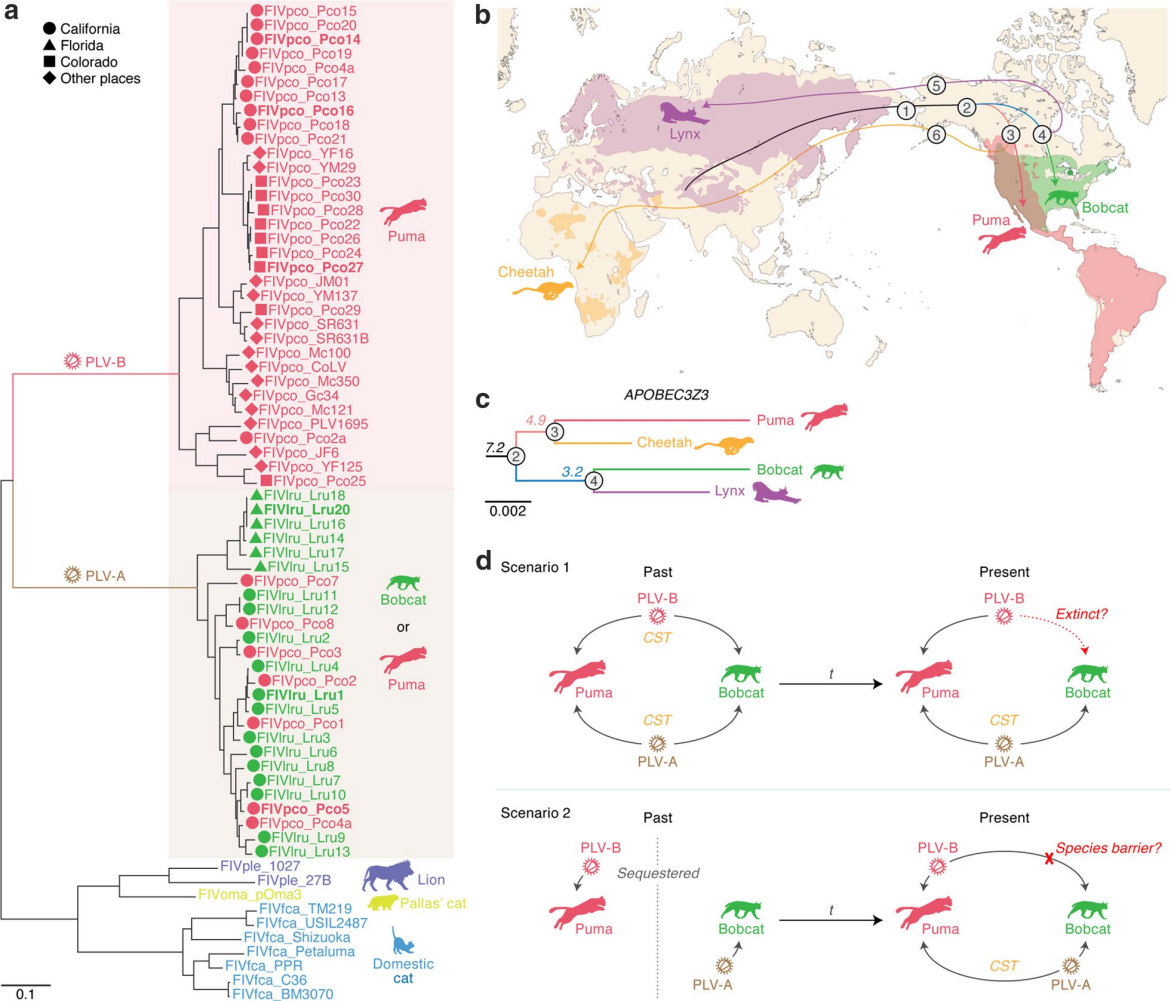
Evolutionary relationship of FIV *vif* and feline *A3Z3.* **a** Phylogenetic tree of FIV *vif.* This phylogenetic tree was constructed using the ML method and displays the evolutionary relationships among the FIV sequences used in this study. The scale bar indicates 0.1 nucleotide substitutions per site. The bootstrap values are indicated on each node as follows: *, > 50 and **, > 80. The FIV *vif* genes used in this study are indicated in bold. The PLV-A and PLV-B sampling locations are available from previous studies [17, 19, 59] and are indicated by symbols. **b**, **c** Evolutionary history of felids in the puma and bobcat lineages. **b** The evolutionary events of the four felid species (puma, bobcat, cheetah and lynx) are summarized according to a previous report [23]. The numbers in circles indicate the events as follows: 1, migration of the common ancestor of the puma (*Puma concolor*) and bobcat (*Lynx rufus*) through the Bering Isthmus from Eurasia to the New World (ca. 8.0–8.5 Mya); 2, divergence into the two lineages (ca. 7.2 Mya); 3, divergence of the puma and cheetah (*Acinonyx jubatus*) (ca. 4.9 Mya); 4, divergence of the bobcat and lynx (*Lynx lynx*) (ca. 3.2 Mya); 5, migration of the lynx from the New World to Eurasia (ca. 1.2–1.6 Mya); and 6, migration of the cheetah from the New World to Eurasia (ca. 1.2–1.6 Mya). The current habitats of the puma (red), bobcat (green), cheetah (yellow) and lynx (purple) are indicated by each color and are referred from the IUCN Red List of Threatened Species website (http://www.iucnredlist.org/). **c** Phylogenetic tree of feline *A3Z3.* The bobcat and cheetah *A3Z3* sequences, which were newly identified in this study, were aligned with those of the puma and lynx, and the tree was reconstructed using the ML method. The branch colors correspond to those of the lines in **b**, and the circled numbers on the nodes correspond to those in **b**. The numbers under nodes in italics indicate the age of divergence (Mya) estimated in a previous study [23]. The scale bar indicates 0.002 nucleotide substitutions per site. **d** Two possible scenarios leading to the inter-species PLV transmission between the puma and bobcat. Each scenario is explained in the main text. *CST* cross-species transmission; *t* time

In addition to FIVfca in domestic cats, various FIV types have been identified in other felids, such as FIVple in lions (*Panthera leo*), FIVpco in pumas (*Puma concolor*), and FIVlru in bobcats (*Lynx rufus*) (reviewed in [16]). Interestingly, although these felids become infected with a specific FIV species, Lee et al. [17] recently reported that a subcluster of FIVpco co-circulated in both pumas and bobcats in North America. The authors designated these viruses (FIVpco and FIVlru) puma lentiviruses (PLVs) and re-classified them as follows: PLV-A includes the FIVpco and FIVlru strains co-circulating in both pumas and bobcats, and PLV-B includes FIVpco strains circulating only in pumas (Fig. 1a) [17–19]. This scenario is the first known case indicating cross-species transmission (CST) of a lentivirus between hosts of different genera (*Puma* and *Lynx*) at present. Since *A3* genes are under positive selection [12, 20–22], the A3 sequences are highly variable among hosts of different genera. Furthermore, we can reasonably hypothesize that the specificity in which Vif counteracts the host A3 also differs in each host genus. These findings and insights raise the hypothesis that Vif-A3 interplay between FIV and the two New World felids (puma and bobcat) is closely associated with the mode of CST among lentiviruses, which may illustrate the history of the co-evolutionary arms race between lentiviruses and felids in the New World.

In this study, we perform cell-based virological experiments and demonstrate that the species specificity of the Vif proteins of PLV-A and PLV-B is different between each other. Through the combinational investigations of experimental virology and molecular phylogenetics, we also provide evidence suggesting that bobcat A3Z3 plays a role as the species barrier specifically against PLV-B. Moreover, we determine the amino acid residue responsible for the sensitivity to PLV-B Vif-dependent degradation. This is the first report addressing the co-evolutionary interplay between antiviral A3 of the two New World felids and feline lentiviral Vif.

## Results

### Bobcat A3Z3 is resistant to PLV-B Vif-mediated degradation

To elucidate the co-evolutionary relationship between PLVs and their host felids, particularly between viral Vif and host A3Z3, first we constructed a phylogenetic tree of FIV *vif* gene. As shown in Fig. 1a, FIVfca and FIVple formed clusters based on their hosts. In sharp contrast, the FIVlru sequences were co-mingled with some FIVpco sequences, whereas the other FIVpco sequences formed a cluster (Fig. 1a). These results are consistent with the phylogeny of the full-length FIV sequences shown in a previous study [17]. According to this previous report, here we designated the FIVlru and co-mingled FIVpco sequences PLV-A and the other FIVpco sequences PLV-B. The sequence identities of PLV-A Vif and PLV-B Vif were 90.1 ± 4.8 and 85.7 ± 7.1%, respectively, whereas the Vif sequence identity between PLV-A and PLV-B was 40.6 ± 1.1%. Thus, the PLV-A Vif and PLV-B Vif sequences clearly differed.

Next, we focused on the host felid evolutionary relationships. Based on comprehensive genetic information, a previous study [23] described the evolutionary history of the felids of interest in this study (Fig. 1b). The common ancestor of the puma (*Puma concolor*) and the bobcat (*Lynx rufus*) is estimated to have crossed the Bering Isthmus from Eurasia to the New World approximately 8.0–8.5 million years ago (Mya) and to have diverged approximately 7.2 Mya [23]. The puma diverged from the cheetah (*Acinonyx jubatus*) approximately 4.9 Mya, whereas the bobcat was branched from the lynx (*Lynx lynx*) approximately 3.2 Mya (Fig. 1b) [23]. Puma and bobcat resided in the New World, whereas lynx and cheetah returned to Eurasia approximately 1.2–1.6 Mya (Fig. 1b) [23]. To assess the phylogenetic relationships of the *A3Z3* genes of these felids, we collected body hairs from a bobcat and a cheetah from Japanese zoos and determined their *A3Z3* sequences (see Additional file 1: Fig. S1 and Additional file 2: Table S1). As shown in Fig. 1c, the topology of the phylogenetic tree of the *A3Z3* genes form these four felids corresponded to their evolutionary relationships.

Based on these observations, two scenarios are assumed. The first scenario was that both PLV-A and PLV-B co-circulated in pumas and bobcats in the New World but PLV-B became extinct only in the bobcat population (“scenario 1” in Fig. 1d). The other possibility was that PLV-A and PLV-B were specific viruses in bobcats and pumas, respectively, in the past. After co-habitation of these two feline species in the New World, PLV-A was transferred from bobcats to pumas, whereas PLV-B CST from pumas to bobcats was hampered for unknown reasons (“scenario 2” in Fig. 1d).

**Fig. 2:**
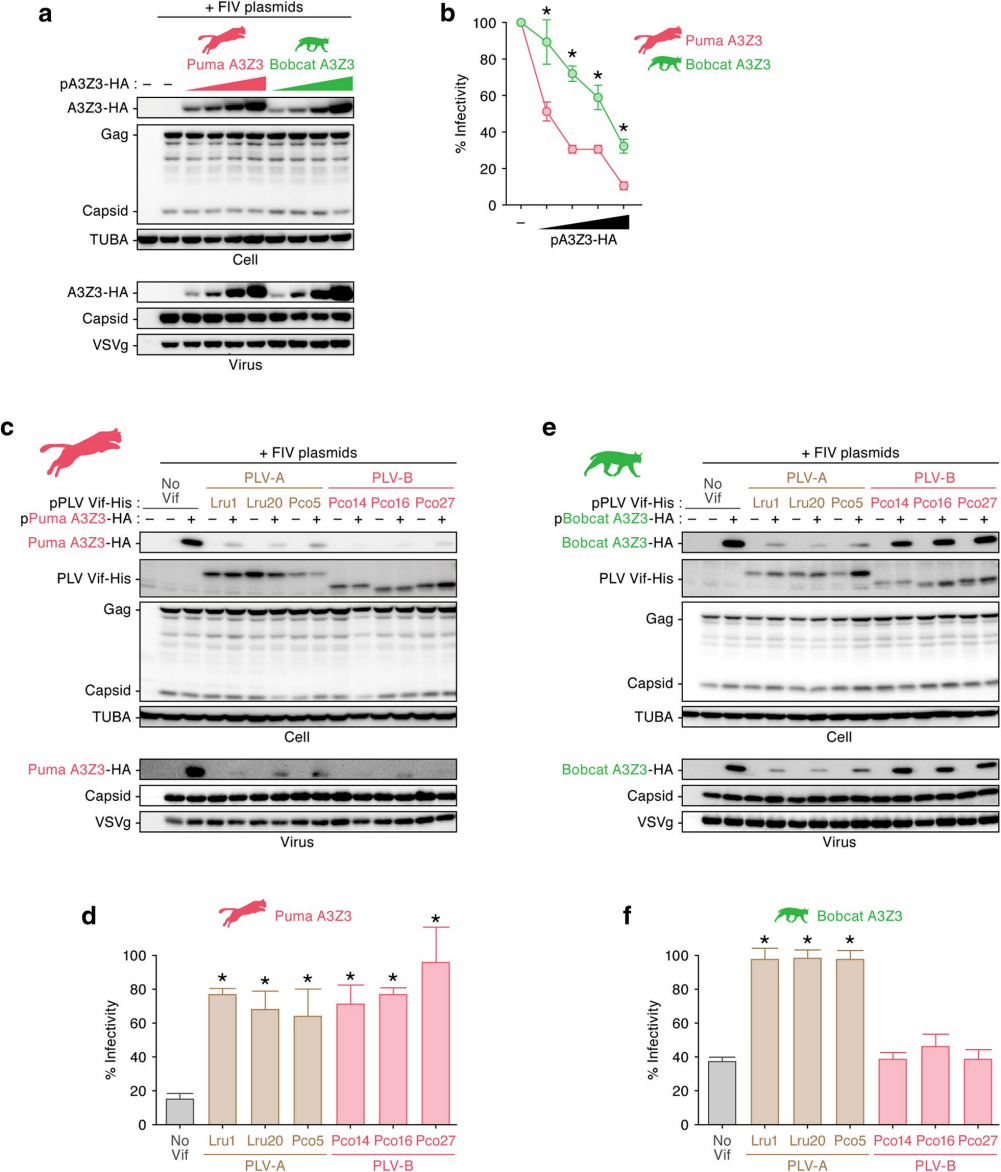
Resistance of bobcat A3Z3 to PLV-B Vif-dependent degradation. **a**, **b** Antiviral effects of the puma and bobcat A3Z3 proteins. Different amounts of HA-tagged expression plasmids for puma or bobcat A3Z3 (0, 50, 100, 200 and 400 ng) and the three plasmids used to produce the *vif*-deficient FIV-based reporter virus (FIV plasmids; pFP93 [200 ng], pTiger-luc [150 ng] and pMD.G [50 ng]) were co-transfected into HEK293T cells. **a** Western blotting. Representative results are shown. **b** FIV reporter assay. FIV infectivity is shown as the percentage of the value of “no A3Z3”. **c**–**f** Puma and bobcat A3Z3 sensitivity to PLV Vif. HA-tagged expression plasmids for puma (**c**, **d**) and bobcat (**e**, **f**) A3Z3 (200 ng) and the three plasmids used to produce the *vif*-deficient FIV-based reporter virus (FIV plasmids; pFP93 [200 ng], pTiger-luc [150 ng] and pMD.G [50 ng]) were co-transfected with or without His-tagged PLV Vif expression plasmids (400 ng) into HEK293T cells. **c**, **e** Western blotting. Representative results are shown. **d**, **f** FIV reporter assay. FIV infectivity is shown as the percentage of the value of “no A3Z3”. In **b**, asterisks indicate significant differences (*P* < 0.05 by Student’s *t* test) between puma A3Z3 and bobcat A3Z3. In **d** and **f**, asterisks indicate significant differences (*P* < 0.05 by Student’s *t* test) versus “no Vif”. The assays were independently performed in triplicate. Data represent averages with SDs

To experimentally investigate the interplay between PLVs and their hosts, we analyzed the antiviral activity of the puma and bobcat A3Z3 proteins. In the absence of PLV Vif, both puma and bobcat A3Z3 were expressed at comparable levels and are incorporated into the released particles of *vif*-deficient FIV (strain Petaluma) in a dose-dependent manner (Fig. 2a). In addition, the infectivity of *vif*-deficient FIV was suppressed by both puma and bobcat A3Z3 proteins in a dose-dependent manner (Fig. 2b). Interestingly, although the protein expression (Fig. 2a, top) and incorporation levels in the released virions (Fig. 2a, bottom) were similar between the puma and bobcat A3Z3 s, the antiviral effect was significantly higher for the puma A3Z3 than for the bobcat A3Z3 (Fig. 2b).

Next, we analyzed the sensitivity of puma and bobcat A3Z3 to PLV Vif proteins. We used expression plasmids for three PLV-A Vif strains (Lru1, Lru20 and Pco5) and three PLV-B Vif strains (Pco14, Pco16 and Pco27). The sampling years and locations for each virus are summarized in Table 1. As shown in Fig. 2c, the puma A3Z3 was degraded by all of the PLV Vif proteins tested in this study, and the antiviral effect of puma A3Z3 was counteracted by these PLV Vif proteins (Fig. 2d). In sharp contrast, the bobcat A3Z3 was degraded by the PLV-A Vif proteins but was resistant to degradation mediated by the PLV-B Vif proteins (Fig. 2e, top). Bobcat A3Z3 was incorporated into nascent virions even in the presence of PLV-B Vif (Fig. 2e, bottom), which significantly decreased viral infectivity (Fig. 2f). Taken together, these findings suggest that bobcat A3Z3 is resistant to PLV-B Vif-mediated degradation.

**Table 1:** PLV Vif used in this study

Group	Strain	Sampling location	Sampling year	Accession nos.
PLV-A	Lru1	California	1996	KF906143
PLV-A	Lru20	Florida	2010	KF906162
PLV-A	Pco5	California	2004	KF906167
PLV-B	Pco14	California	2002	KF906182
PLV-B	Pco16	California	2011	KF906193
PLV-B	Pco27	Colorado	2008	KF906194

### Puma A3Z3 is uniquely sensitive to PLV-B Vif-mediated degradation

**Fig. 3:**
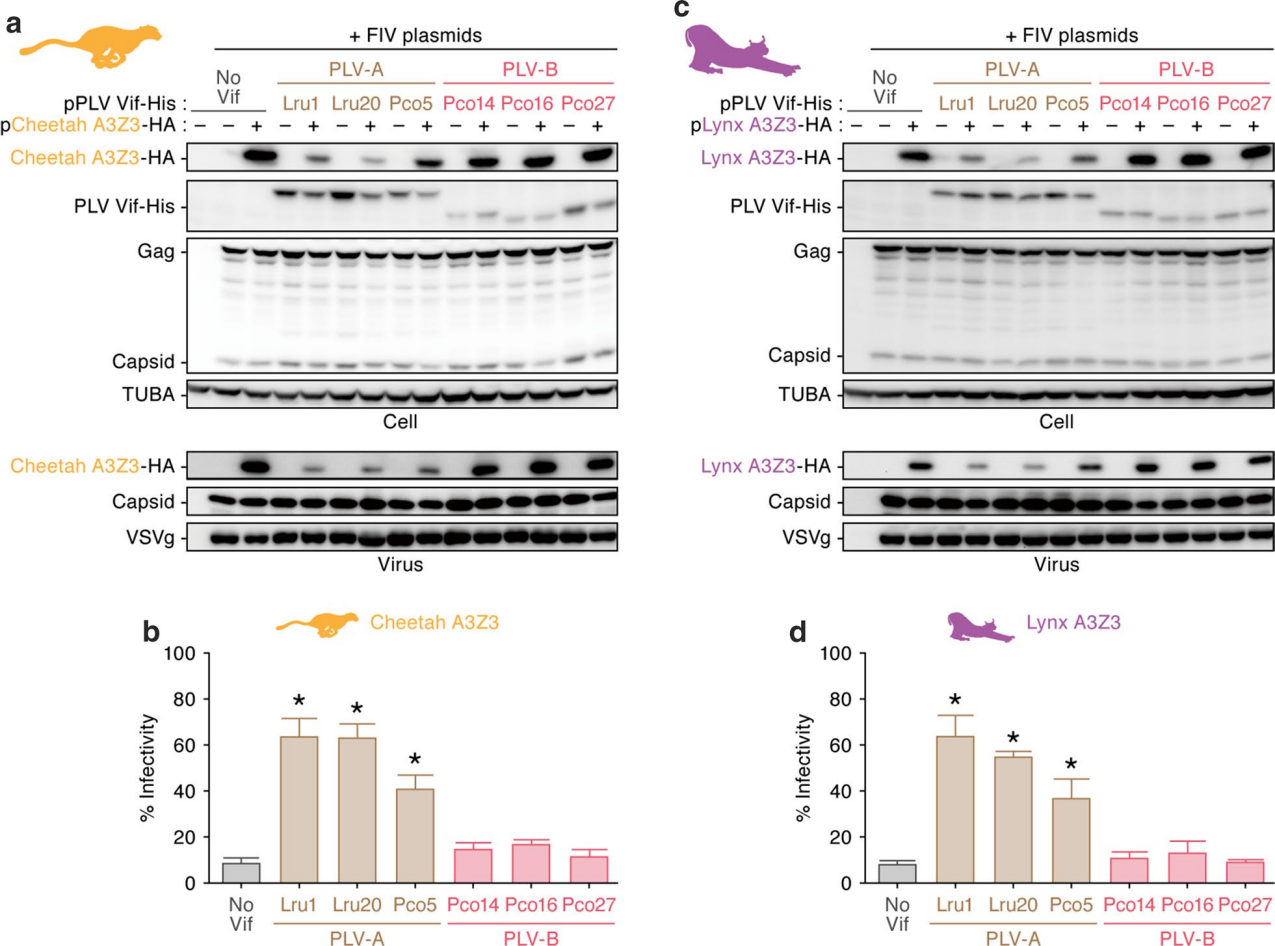
A3Z3 sensitivity of the felids related to pumas and bobcats to PLV Vif-mediated degradation. HA-tagged expression plasmids for cheetah (**a**, **b**) and lynx (**c**, **d**) A3Z3 (200 ng) and the three plasmids used to produce the *vif*-deficient FIV-based reporter virus (FIV plasmids; pFP93 [200 ng], pTiger-luc [150 ng] and pMD.G [50 ng]) were co-transfected with or without His-tagged PLV Vif expression plasmids (400 ng) into HEK293T cells. **a**, **c** Western blotting. Representative results are shown. **b**, **d** FIV reporter assay. FIV infectivity is shown as the percentage of the value of “no A3Z3”. In **b** and **d**, asterisks indicate significant differences (*P* < 0.05 by Student’s *t* test) versus “no Vif”. The assays were independently performed in triplicate. Data represent averages with SDs

As shown in Fig. 1b, the puma and bobcat are evolutionarily similar to the cheetah (*Acinonyx jubatus*) and the lynx (*Lynx lynx*), respectively. To address whether the sensitivity of host A3Z3 to PLV-B Vif is associated with the host phylogeny, we constructed expression plasmids for cheetah and lynx A3Z3 proteins and performed similar experiments. Our results revealed that both cheetah and lynx A3Z3 were degraded by PLV-A Vif but were resistant to PLV-B Vif-mediated degradation (Fig. 3a, b, top). Similar to bobcat A3Z3, the cheetah and lynx A3Z3 proteins were incorporated into the released virions even in the presence of PLV-B Vif (Fig. 3a, b, bottom) resulting in an antiviral effect (Fig. 3c, d). These findings suggest that the PLV-A Vif antagonizes all four feline A3Z3 tested in this study, whereas PLV-B Vif antagonizes only the puma A3Z3.

### The threonine residue at position 178 of bobcat A3Z3 confers the resistance to PLV-B Vif-mediated degradation

**Fig. 4:**
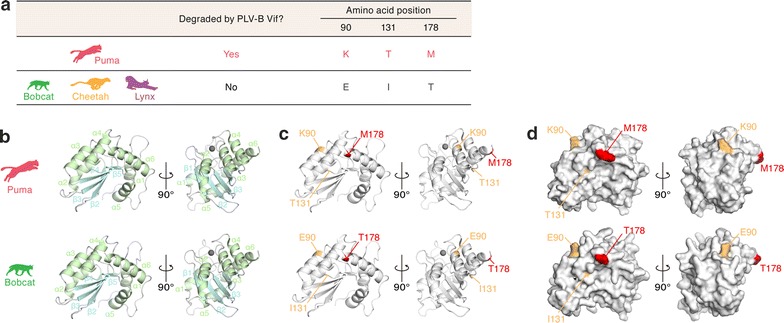
Structure modeling of puma and bobcat A3Z3. **a** Comparison of amino acid residues. The feline species used for the comparison (puma, bobcat, cheetah and lynx; left), the sensitivity of A3Z3 to PLV-B Vif (middle) and the amino acid residues at positions 90, 131 and 178 (right) are summarized. **b**–**d** Structural homology model of puma and bobcat A3Z3. Cartoon (**b**, **c**) and surface (**d**) models of the A3Z3 protein structures of the puma (top) and bobcat (bottom) are shown. In **b**, alpha-helices and beta-sheets are shown in green and pale blue, respectively. In the right panels of **a** and **b**, Zn^2+^ is represented as a gray sphere. In **c** and **d**, the three amino acids that differed between the puma and the other felids shown in **a** (bobcat, cheetah and lynx; residues 90, 131 and 178) are represented in orange or red

We compared the amino acid sequence of puma, bobcat, cheetah and lynx A3Z3s and found three amino acids at positions 90, 131 and 178 that consistently differed between the puma and the other felids (Fig. 4a). To assess the positions of these three residues in the tertiary structures of the puma and bobcat A3Z3 proteins, we constructed A3Z3 protein homology models for these felids (Fig. 4b). Residues 90 and 178 are located in alpha-helices 3 and 6, respectively, whereas residue 131 is positioned in the loop (Fig. 4c). Additionally, residues 90 and 178 are localized on the protein surface (Fig. 4d).

**Fig. 5:**
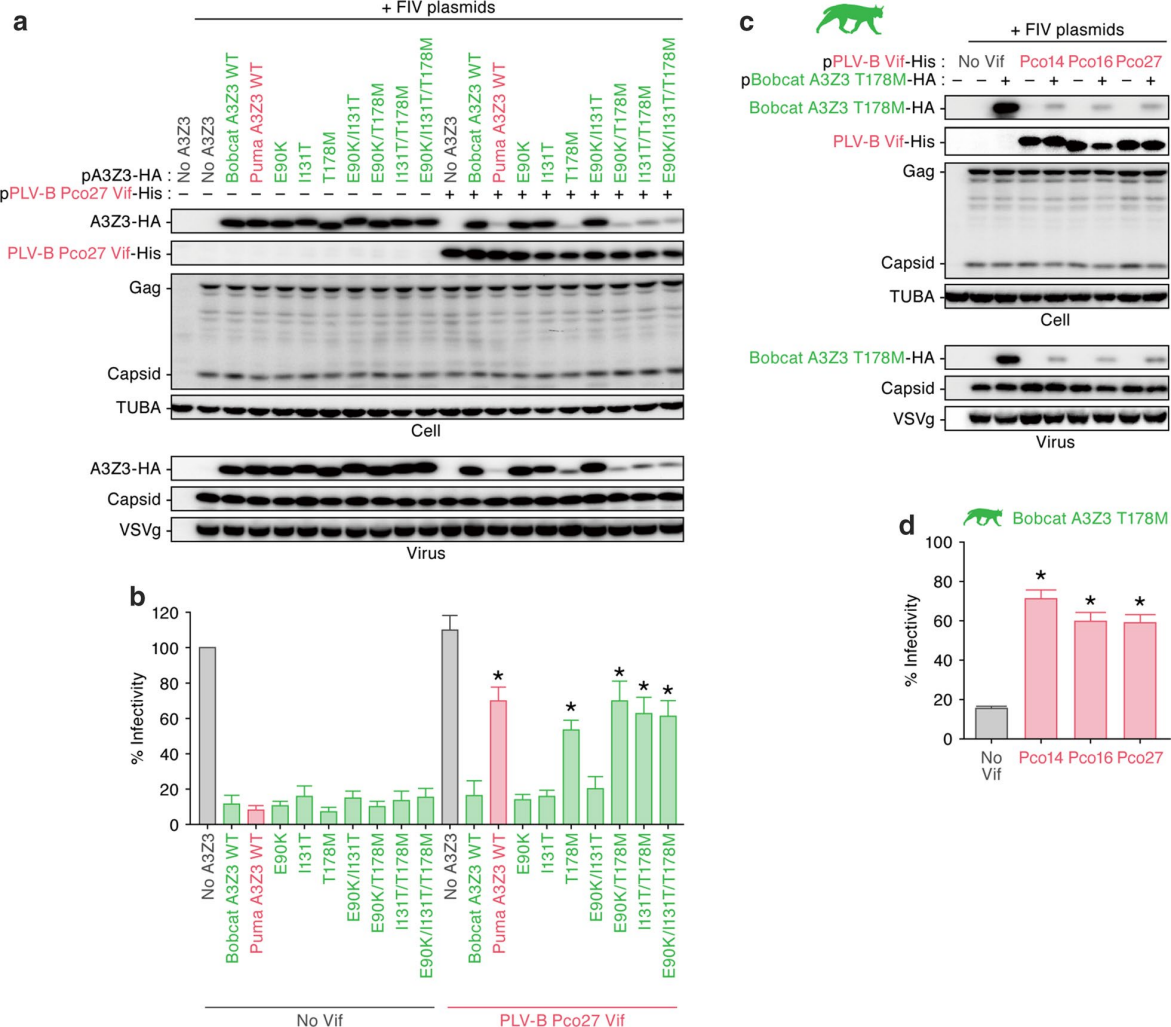
Loss-of-function screening of the amino acid residues responsible for PLV-B Vif sensitivity. HA-tagged expression plasmids for the A3Z3 derivatives (indicated in the figure) of the bobcat (200 ng) and the three plasmids used to produce the *vif*-deficient FIV-based reporter virus (FIV plasmids; pFP93 [200 ng], pTiger-luc [150 ng] and pMD.G [50 ng]) were co-transfected with or without His-tagged PLV-B Vif expression plasmids (400 ng) into HEK293T cells. **a**, **c** Western blotting. Representative results are shown. **b**, **d** FIV reporter assay. FIV infectivity is shown as the percentage of the value of “no A3Z3”. In **b** and **d**, asterisks indicate significant differences (*P* < 0.05 by Student’s *t* test) versus “no Vif”. The assays were independently performed in triplicate. Data represent averages with SDs

To determine the amino acid residue(s) responsible for the resistance to PLV-B Vif-mediated degradation, we constructed lines of bobcat A3Z3 mutants and performed cell-based loss-of-function experiments. Although the bobcat A3Z3 E90K and I131T mutants were resistant to PLV-B Vif-mediated degradation (strain Pco27), the bobcat A3Z3 T178M mutant was degraded by PLV-B Vif (Fig. 5a). We also used combination mutants and found those harboring the T178M substitution lost the ability to resist PLV-B Vif-mediated degradation (strain Pco27) (Fig. 5a). In the presence of PLV-B Vif, incorporation of bobcat A3Z3 derivatives possessing the T178M mutation into the released viruses was impaired (Fig. 5a, bottom), and the derivatives’ antiviral effects were abrogated (Fig. 5b). To determine whether this observation was strain-specific, we performed similar experiments using the other PLV-B strains (Pco14 and Pco16). Similar to strain Pco27 (Fig. 5a, b), the Vif proteins of the other PLV-B strains degraded the bobcat A3Z3 T178M mutant (Fig. 5c) and significantly recovered viral infectivity (Fig. 5d). These findings suggest that the resistance of bobcat A3Z3 to PLV-B Vif-mediated degradation is determined by the amino acid residue at position 178.

**Fig. 6:**
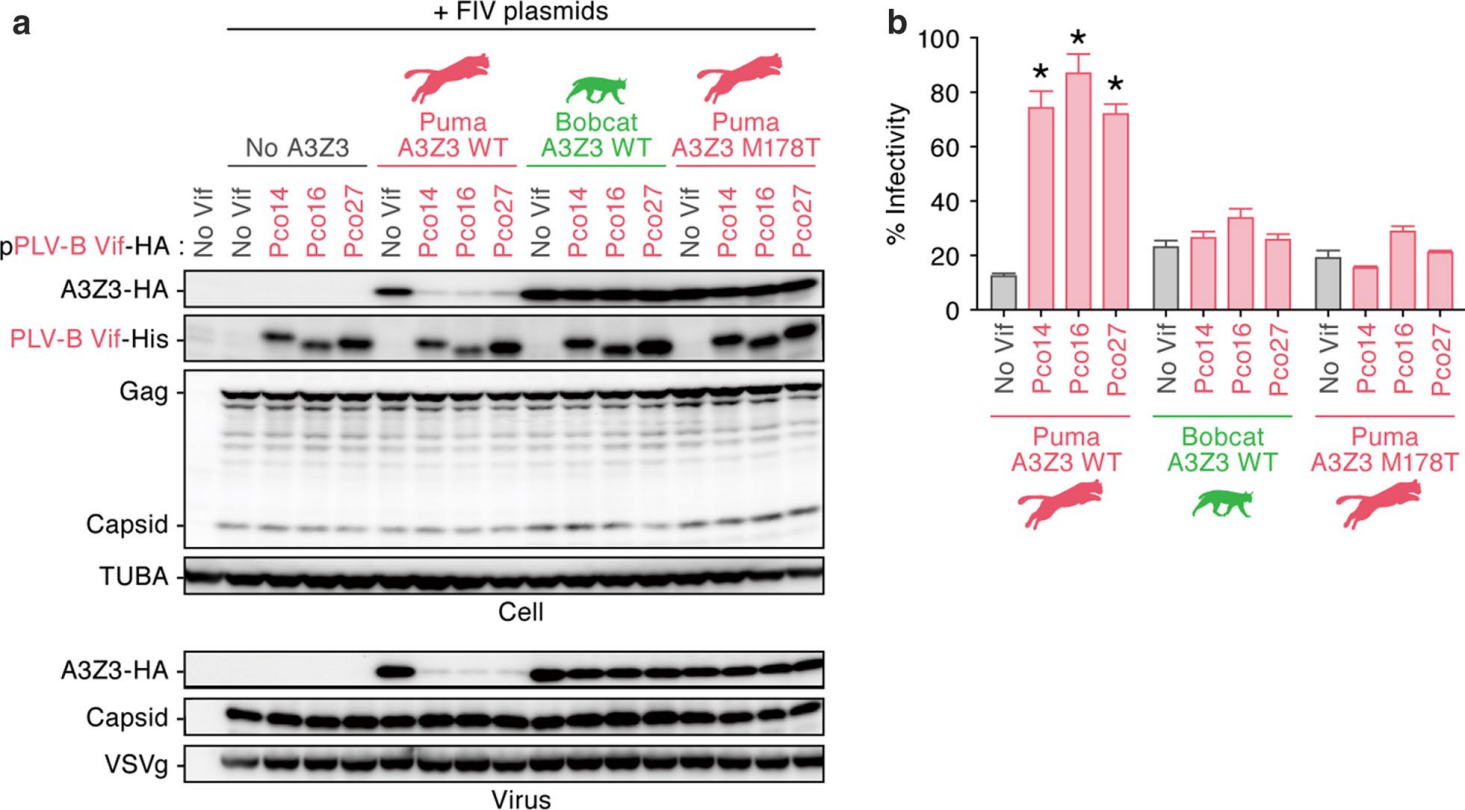
Gain-of-function validation of the amino acid residues responsible for PLV-B Vif sensitivity. HA-tagged expression plasmids for puma A3Z3, bobcat A3Z3 and the puma A3Z3 M178T derivative (200 ng) and the three plasmids used to produce *vif*-deficient FIV-based reporter virus (FIV plasmids; pFP93 [200 ng], pTiger-luc [150 ng] and pMD.G [50 ng]) were co-transfected with or without His-tagged PLV-B Vif expression plasmids (400 ng) into HEK293T cells. **a** Western blotting. Representative results are shown. **b** FIV reporter assay. FIV infectivity is shown as the percentage of the value of “no A3Z3”. In **b**, asterisks indicate significant differences (*P* < 0.05 by Student’s *t* test) versus “no Vif”. The assays were independently performed in triplicate. Data represent averages with SDs

To validate the importance of the amino acid at position 178 to sensitivity to PLV-B Vif-mediated degradation, we performed gain-of-function experiments based on the puma A3Z3. By substituting the methionine residue at position 178 of the puma A3Z3 to threonine, the puma A3Z3 mutant became resistant to PLV-B Vif-mediated degradation (Fig. 6a, top). The puma A3Z3 M178T mutant was efficiently incorporated into the released virions (Fig. 6a, bottom) and exhibited significant antiviral activity (Fig. 6b). Taken together, these findings suggest that the amino acid at position 178 in the bobcat/puma A3Z3 plays a pivotal role in conferring resistance to PLV-B Vif-mediated degradation.

### Puma and bobcat A3Z3 are polymorphic

**Fig. 7:**
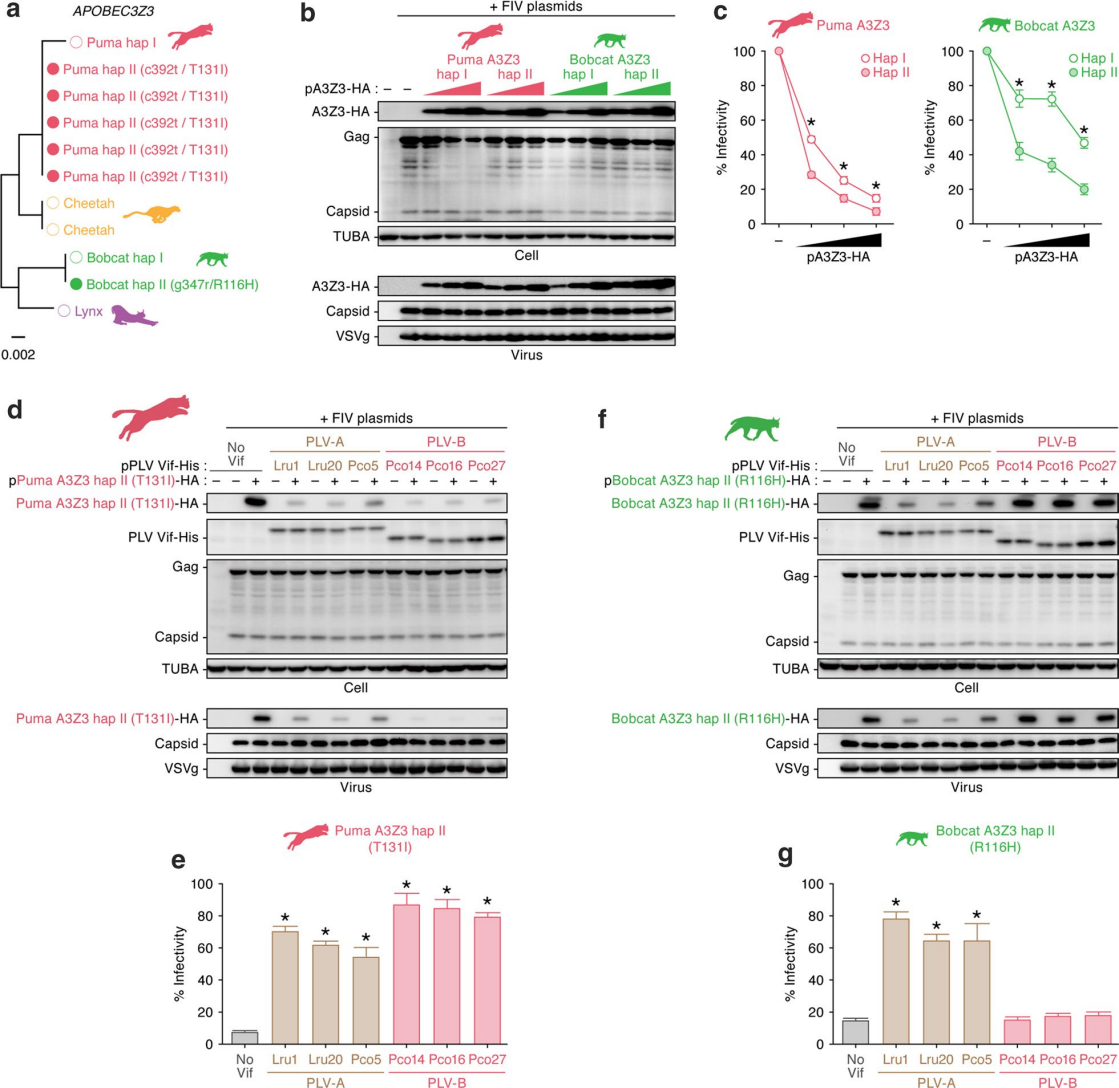
Polymorphisms of puma and bobcat *A3Z3* and their association with PLV Vif sensitivity. **a** Feline *A3Z3* polymorphisms. Additional *A3Z3* sequences from five pumas, one cheetah and one bobcat were determined and the phylogenetic tree was reconstructed using the ML method. The *A3Z3* sequences used in the experiments above (summarized in Fig. 1c) are indicated with open circles and designated hap I. Additional *A3Z3* haplotypes detected in the puma and bobcat are indicated with filled circles and designated hap II. The nucleotide/amino acid substitutions detected in puma and bobcat A3Z3 hap II are indicated in parentheses. Note that the new cheetah *A3Z3* sequence was identical to the previously determined sequence (Fig. 1c). The scale bar indicates 0.002 nucleotide substitutions per site. **b**–**f** Antiviral activity of the puma and bobcat A3Z3 hap II proteins and their sensitivity to PLV Vif. **b**, **c** Different amounts of HA-tagged expression plasmids for A3Z3 hap I and II of puma or bobcat (0, 100, 200 and 400 ng) and the three plasmids used to produce the *vif*-deficient FIV-based reporter virus (FIV plasmids; pFP93 [200 ng], pTiger-luc [150 ng] and pMD.G [50 ng]) were co-transfected into HEK293T cells. **b** Western blotting. Representative results are shown. **c** FIV reporter assay. FIV infectivity is shown as the percentage of the value of “no A3Z3”. Note that “hap I” is identical to those used in the other experiments shown in Figs. 1, 2, 4, 5 and 6. **d**–**g** HA-tagged expression plasmids for puma A3Z3 hap II (**d**, **e**) or bobcat A3Z3 hap II (**f**, **g**) (200 ng) and the three plasmids used to produce the *vif*-deficient FIV-based reporter virus (FIV plasmids; pFP93 [200 ng], pTiger-luc [150 ng] and pMD.G [50 ng]) were co-transfected with or without His-tagged PLV Vif expression plasmids (400 ng) into HEK293T cells. **d**, **f** Western blotting. Representative results are shown. **e**, **g** FIV reporter assay. FIV infectivity is shown as the percentage of the value of “no A3Z3”. In **b**, asterisks indicate significant differences (*P* < 0.05 by Student’s *t* test) between hap I and hap II. In **e** and **g**, asterisks indicate significant differences (*P* < 0.05 by Student’s *t* test) versus “no Vif”. The assays were independently performed in triplicate. Data represent averages with SDs

Previous studies have revealed that mammalian *A3* genes, including feline *A3Z3* genes, are under positive selection and are highly diversified due to evolutionary selective pressures presumably caused by the ancestral FIV Vif [23]. For instance, the domestic cat *A3Z3* is polymorphic [24], and a haplotype of the domestic cat A3Z3 renders resistance to FIVfca Vif-mediated degradation [12]. These previous findings raise the possibility that the puma and bobcat *A3Z3* genes are also polymorphic and that the PLV Vif sensitivity may differ among haplotypes. To address this possibility, we additionally determined the *A3Z3* sequences of five pumas, one bobcat as well as one cheetah. As shown in Fig. 7a, we detected additional puma and bobcat *A3Z3* haplotypes and designated them haplotype II (hap II). One nonsynonymous mutation was detected in the puma A3Z3 hap II (a c392t mutation resulting in T131I amino acid substitution), and a heterozygous sequence was detected in the bobcat A3Z3 hap II (g347r; a g347a mutation resulting in a R116H amino acid substitution) (Fig. 7a). Next, we mapped these residues on the protein homology model. Both residue 131 of the puma A3Z3 (Fig. 4b) and residue 116 of the bobcat A3Z3 (Additional file 3: Fig. S2) are positioned in the loop structure. Then, we prepared expression plasmids for these haplotypes and assessed their antiviral activity and sensitivity to PLV Vif. As shown in Fig. 7b, puma and bobcat A3Z3 hap II were expressed at similar levels to their hap I derivatives. Notably, in the absence of PLV Vif, the antiviral effects of the puma and bobcat A3Z3 hap II were significantly higher than those of the hap I derivatives (Fig. 7c). In particular, the bobcat A3Z3 hap II exhibited stronger antiviral activity in a dose-dependent manner (Fig. 7c). Moreover, puma A3Z3 hap II was sensitive to both the PLV-A and PLV-B Vifs (Fig. 7d, e), whereas the bobcat A3Z3 hap II was sensitive to PLV-A Vif but was resistant to PLV-B Vif-mediated degradation (Fig. 7e, f). Taken together, these findings suggest that the puma and bobcat *A3Z3* genes are polymorphic and that the bobcat A3Z3 hap II exhibits a higher antiviral effect than the hap I derivative, whereas the PLV Vif sensitivity phenotype is conserved within each species.

## Discussion

In this study, we identified the *A3Z3* sequences of two felids, the bobcat and cheetah, and demonstrated that PLV-A Vif counteracted the A3Z3 proteins of both the puma and bobcat lineages, whereas PLV-B Vif counteracted only the puma A3Z3 (Figs. 1, 2, 3). Through loss-of-function (Fig. 5) and gain-of-function (Fig. 6) experiments, we also determined that the amino acid at position 178 of the puma and bobcat A3Z3 was responsible for the sensitivity to PLV-B Vif. In addition, structural modeling suggested that this residue was exposed to the protein surface (Fig. 4). Furthermore, although the puma and bobcat *A3Z3* genes were polymorphic, the sensitivity to PLV-B Vif was conserved in each species (Fig. 7). Some previous studies have addressed the potential of OWM A3G to restrict HIV-1 infection [25, 26]. However, OWMs do not infect with HIV-1 in nature. Therefore, to the best of our knowledge, this study is the first to suggest that inter-species lentiviral transmission in nature is controlled by a host A3 protein.

As summarized in Fig. 1a, d, PLV-A is shared by both pumas and bobcats in North America, whereas PLV-B is detected only in pumas. Through cell-based virological experiments, here we demonstrated that PLV-A Vif degraded the A3Z3 protein of feline lineages including puma and bobcat, whereas PLV-B Vif counteracted only the puma A3Z3. Additionally, the bobcat A3Z3 exhibited a significant antiviral effect even in the presence of PLV-B Vif. Because the functionality of PLV Vif is independent of the sampling location and year (Fig. 2 and Table 1), our results suggest that the functional relationship between PLV Vif and feline A3Z3 is conserved. Moreover, we found a polymorphism in the puma *A3Z3*, but the sensitivity of the puma A3Z3 variants to PLV-B Vif-mediated degradation was also conserved (Fig. 6). These findings suggest that “scenario 2” reasonably explains the difference in species tropism between PLV-A and PLV-B and that bobcat A3Z3 acts as a species barrier to restrict PLV-B CST of PLV-B from pumas (Fig. 1d). To the best of our knowledge, this is the first report demonstrating that the host A3 protein is the factor that restricts non-primate lentiviral CST.

Consistent with our results, PLV-A and PLV-B form clusters by sampling location [17–19]. Moreover, the PLV-As detected in pumas (FIVpco) and bobcats (FIVlru), particularly those isolated in California, were highly co-mingled in a cluster (Fig. 1a). These observations suggest that PLV-As have been transmitted relatively recently from bobcats to pumas after geological sequestration, and we can assume that this bobcat-to-puma CST event occurs frequently at least in California.

Our findings in New World felids were reminiscent of the fact that simian immunodeficiency viruses (SIVs) in Old World monkeys (OWMs) were transferred to chimpanzees, which originated in the emergence of a novel SIV in chimpanzees (SIVcpz) (reviewed in [27, 28]). SIV CST from OWMs to chimpanzees in Africa is explained phylogenetically and is assumed to be due to the prey-predator relationship between OWMs and chimpanzees. Small OWMs are the prey of chimpanzees in the wild; thus, chimpanzees are frequently exposed to various SIVs that infect their OWM prey species [29, 30]. Similarly, pumas are a top carnivore in the New World [31] and bobcats are their prey [32, 33]. Therefore, pumas may be frequently exposed to PLV-A in bobcats via prey-predator interactions, leading to bobcat-to-puma CST; this possibility is consistent with a previous assumption based on phylogenetic analyses [17].

Although PLV-A Vif degraded all of the feline A3Z3 proteins tested in this study, PLV-B Vif counteracted only the puma A3Z3, suggesting the evolutionary convergence of the puma and PLV-B. Compton and Emerman reported that the Vif protein of an SIV infecting colobus monkey (*Colobus guereza*), which is an OWM in Africa, counteracted only colobus A3G [34]. Based on findings for the A3G-Vif interaction in colobus monkeys, the authors proposed convergent evolution of colobus monkeys and SIVcor and further suggested that the convergent co-evolution of these monkeys and viruses occurred at least 12 Mya [34]. Similarly, the puma diversified from the cheetah approximately 4.9 Mya (Fig. 1c) [23]. Puma and cheetah habitats were separated approximately 1.2–1.6 Mya after cheetahs immigrated from the New World to Eurasia (Fig. 1b) [23]. Since the cheetah A3Z3 was not counteracted by the PLV-B Vif (Fig. 3), our findings suggested that the co-evolutionary convergence of the puma and PLV-B arose at least 1.2–1.6 Mya. Moreover, OWMs exhibit higher viral RNA loads without any disorders [35]. Since SIVs circumvent the immune pressure from host monkeys because they are naturally infected, SIVs are less diversified in African OWMs, which is assumed to be due to the co-existence of SIVs in OWMs over a long period [35]. Lee et al. [17] recently showed that the PLV-B plasma viral RNA load in pumas was significantly higher than PLV-A loads in both pumas and bobcats, whereas PLV-B was less diversified than PLV-A. This is the first study suggesting the convergent co-evolution of a mammal (puma) and a lentivirus (PLV-B) in the New World, and PLV-B can be concluded to have co-evolved with pumas over a long period, similar to apathogenic SIV infections in OWMs.

Through mutagenesis experiments, we demonstrated that the amino acid at position 178 determined the sensitivity to PLV-B Vif-dependent degradation (Fig. 5). Since both the gain- and loss-of-function experiments indicated the importance of this residue, the species specificity of PLV-B Vif against puma and bobcat A3Z3 was determined by this single amino acid. In addition, the structural biology investigation revealed that this amino acid residue was exposed on the A3Z3 protein surface (Fig. 4). In our previous study [12], we revealed that there are at least seven haplotypes in the domestic cat *A3Z3* and that a domestic cat A3Z3 haplotype (designated haplotype V in our previous study) was resistant to FIVfca Vif-mediated degradation. Furthermore, we demonstrated that amino acid residue 65 of the domestic cat A3Z3 determines the sensitivity to FIVfca Vif [12]. Similar to this study, residue 65 in the domestic cat A3Z3 was also exposed on the protein surface, which suggested that this residue was associated with the interaction with FIV Vif [12]. However, the amino acid residue at position 65 was positioned at alpha-helix 2 in the domestic cat A3Z3 [12], whereas the residue 178 was located at alpha-helix 6 in the puma and bobcat A3Z3 (Fig. 5). Therefore, our findings suggest that these residues render resistance to FIV Vif in a different manner.

In addition to A3, other cellular proteins such as tripartite motif containing protein 5 (TRIM5) [36] and tetherin [37, 38] are known as restriction factors that inhibit lentiviral replication in primates. Therefore, it might be possible that these restriction factors in pumas and/or bobcats may also play roles in preventing FIV CST. However, feline TRIM5 is not functional because of the insertion of premature stop codon [39], and feline tetherin is unable to restrict spreading FIV infection [40]. Furthermore, although FIV Vif is a functional antagonist against feline A3, FIV lacks functional proteins that potently counteract TRIM5 and tetherin [39, 40]. Primates encode several restriction factors against lentiviruses; however, only A3 is convincing to be a restriction factor in felids. Therefore, feline A3 may play a pivotal role in controlling CST in felids.

In summary, we demonstrated that bobcat A3Z3 was resistant to PLV-B Vif-dependent degradation, whereas PLV-A Vif overcame the antiviral action mediated by both the puma and bobcat A3Z3. The co-evolutionary relationship between primate A3 proteins and their lentiviral Vifs has been rigorously investigated [20, 22, 34, 41–44]; however, few studies, including ours, have addressed the evolutionary dynamics of non-primate A3 and non-primate lentiviruses [4, 12, 14]. Here, we provided evidence suggesting that lentiviral CST between different genera (*Puma* and *Lynx*) was controlled by the Vif-A3 interaction. To the best of our knowledge, this is the first report providing evidence of the co-evolutionary arms race between mammals and lentiviruses in the New World.

## Methods

### Ethics statement

To determine the feline *A3Z3* sequences, blood, body hair or cryopreserved muscle tissue of pumas, bobcats and cheetahs were kindly provided by the following facilities: Tennoji zoo, Osaka, Japan; Kobe City Oji zoo, Hyogo, Japan; Omoriyama zoo, Akita, Japan; Tama Zoological Park, Tokyo, Japan; Izu Animal Kingdom, Shizuoka, Japan; and Shizuoka Municipal Nihondaira zoo, Shizuoka, Japan. Sampling was performed in accordance with the guideline of Tokyo University of Agriculture, Japan. All experimental protocols were approved by a committee at Tokyo University of Agriculture, Japan.

### Sequencing PCR of the feline *A3Z3* genes

Sequencing PCR of feline *A3Z3* was performed as previously described [45, 46]. Briefly, genomic DNA was extracted from the samples described above using the DNA Extractor FM kit (Wako) or the DNeasy Blood & Tissue it (Qiagen). PCR was performed using the PrimeSTAR GXL DNA polymerase (Takara) and the primers are listed in Additional file 2: Table S1. The obtained PCR products were purified by gel extraction using the QIAquick gel extraction kit (Qiagen). The nucleotide sequences were determined by a DNA sequencing service (Fasmac, Kanagawa, Japan) and the data were analyzed using Sequencher v5.1 software (Gene Codes Corporation).

### Molecular phylogenetic analysis of the FIV *vif* and feline *A3Z3* genes

Molecular phylogenetic analyses were performed as previously described [12, 14, 45–47]. Briefly, the sequences of PLV Vif and feline *A3Z3*, some of which were newly identified in this study (Figs. 1a, c, 7a), were aligned using ClustalW implemented in MEGA7 [48]. The alignments were verified manually at the amino acid level. Phylogenetic trees (Figs. 1a, c, 7a) were reconstructed using the maximum likelihood (ML) method with PhyML [49]. To calculate the amino acid sequence diversity of PLV Vif, 26 and 34 amino acid sequences from PLV-A and PLV-B Vif (summarized in Fig. 1a) are used, respectively. A multiple alignment was generated using L-INS-i in MAFFT [50]. The gapped regions were removed using trimAl with the nogaps option [51], and 227 amino acid sites were used for the analysis. Then, we performed pairwise comparisons of 60 amino acid sequences to calculate the amino acid sequence identity using MEGA7 [48].

### Plasmid construction

The expressing plasmids for HA-tagged puma and lynx A3Z3 were kindly provided by Dr. Carsten Münk [15]. The expressing plasmids for HA-tagged bobcat and cheetah A3Z3 were constructed by using the genomic DNA fragments and the primers listed in Additional file 2: Table S1. The point mutants of HA-tagged puma and bobcat A3Z3 were constructed by using a GeneArt site-directed mutagenesis system (Thermo Fisher Scientific). Each wild-type plasmid was used as the template, and the primers used are listed in Additional file 2: Table S1. The His-tagged PLV-A Vif (strains Lru1, Lru20 and Pco5) and PLV-B (strains Pco14, Pco16 and Pco27) were obtained from GeneArt gene synthesis service (Thermo Fisher Scientific). The obtained DNA fragments were digested with BamHI and SalI and inserted into the BamHI-SalI site of pDON-AI plasmid (Takara). The nucleotide sequences were determined by a DNA sequencing service (Fasmac, Kanagawa, Japan) and the data were analyzed by Sequencher v5.1 software (Gene Codes Corporation).

### Cell culture and transfection

HEK293T cells (CRL-11268; ATCC) were cultured in Dulbecco’s modified Eagle medium (Sigma-Aldrich) supplemented with 10% heat-inactivated fetal calf serum and antibiotics (Thermo Fisher Scientific). Transfection was performed by using PEI Max (GE Healthcare) in accordance with the manufacturers procedures and described previously [12, 14, 45–47, 52]. To analyze the dose-dependent anti-FIV activity of feline A3Z3, pFP93 (pFIVgagpolΔvif; a replication incompetent *vif*-deficient FIV packaging construct derived from clone FIV-34TF10 [GenBank accession number M25381]; kindly provided by Dr. Eric M. Poeschla) (200 ng), pTiger-luc (pFIVΨ-luc) (150 ng), and pMD.G (pVSVg; a vesicular stomatitis virus G [VSVg] expression plasmid) (50 ng) were co-transfected into HEK293T cells (1 × 10^5^ cells) with feline A3Z3 expression plasmid (50, 100, 200, or 400 ng). To analyze the functional relationship between feline A3Z3 and PLV-Vif, feline A3Z3 expression plasmid (200 ng), pFP93 (200 ng), pTiger-luc (150 ng), and pMD.G (50 ng) were co-transfected into HEK293T cells with or without His-tagged PLV Vif expression plasmid (400 ng). After 48 h post-transfection, the transfected cells and culture supernatants were harvested as previously described [12–14, 45–47, 52].

### Western blotting and virus reporter assay

Western blotting and reporter assay were performed as previously described [12–14, 45–47, 52]. For the Western blotting of virus particles, 340 μl of the culture supernatant was ultracentrifuged at 100,000×*g* for 1 h at 4 °C using a TL-100 instrument (Beckman), and the pellet was lysed with 1 × SDS buffer. For the Western blotting of transfected cells, the cells were lysed with RIPA buffer (50 mM Tris–HCl buffer [pH 7.6], 150 mM NaCl, 1% Nonidet P-40, 0.5% sodium deoxycholate, 0.1% SDS) with protease inhibitor cocktail (Roche). The following antibodies for Western blotting: anti-His polyclonal antibody (OGHis; Medical and Biological Laboratories), anti-HA antibody (3F10; Roche), anti-FIV p24 Capsid antibody (PAK3-2C1; Santa Cruz Biotechnology); anti-alpha-tubulin (TUBA) antibody (DM1A; Sigma), and anti-VSVg antibody (P5DA; Roche). For FIV reporter assays, HEK293T cells were used for the target of infection. Ten microliter of the culture supernatant of transfected cells was inoculated into HEK293T cells in a 96-well plate (Nunc), and the firefly luciferase activity was measured by using the BrillianStar-LT assay system (Toyo-b-net) and the 2030 ARVO X multilabel counter instrument (PerkinElmer) according to the manufacturers’ procedures.

### Protein homology modeling

Homology modeling was performed using the SWISS-MODEL server [53–56]. After BLAST searches [57] of the bobcat and puma A3Z3 amino acid sequences against protein data bank sequence entries (http://www.rcsb.org/pdb/), the crystal structure of human APOBEC3A (PDB: 5KEG) [58] was selected as the best template for homology modeling per the Global Model Quality Estimations, QMEAN statistical parameters, and modeled sequence length. Each generated model was minimized and refined using Discovery Studio (Dassault Systèmes BIOVIA, Discovery Studio Modeling Environment, Release 4.1, San Diego: Dassault Systèmes, 2007). Mutant models and 3D images were generated with PyMOL (The PyMOL Molecular Graphics System, version 1.8 Schrödinger, LLC).

### Statistical analyses

The data are expressed as averages with the standard deviations (SDs), and statistically significant differences were determined using Student’s *t* test.

### Accession numbers

The *A3Z3* sequences of five pumas, two bobcats and two cheetahs were submitted to DDBJ (accession numbers LC376039-LC376042).

## Additional files

**Additional file 1: Figure S1.** Scheme of the feline genome encoding *APOBEC3Z3* and the position of the primers used in this study. The scheme used for *Felis catus* chromosome B4, including the exons of feline *APOBEC3Z3,* is shown. The primers used for PCR/sequencing are shown as red arrowheads, and the names are identical to those in Table S1.

**Additional file 2: Table S1.** Primers used in this study. A full list of the primers used in this study.

**Additional file 3: Figure S2.** Structure homology model of bobcat A3Z3 hap II. Cartoon (top and middle) and surface (bottom) models of the A3Z3 protein structures of bobcat hap I (A) and hap II (B) are shown. In the top panel, alpha-helices and beta-sheets are shown in green and pale blue, respectively. Zn^2+^ is represented as a gray sphere. In the middle and bottom panels, the amino acid that differed between hap I (R116) and hap II (H116) is represented in orange.

## Abbreviations

A3: apolipoprotein B mRNA-editing enzyme catalytic polypeptide-like 3 (APOBEC3)
A3Z3: APOBEC3Z3
CST: cross-species transmission
FIV: feline immunodeficiency virus
ML: maximum likelihood
Mya: million years ago
OWM: Old World monkey
PLV: puma lentivirus
SIV: simian immunodeficiency virus
SD: standard deviation
TRIM5: tripartite motif containing protein 5
TUBA: alpha-tubulin
Vif: viral infectivity factor
VSVg: vesicular stomatitis virus G
