# Supplementary figures and images for: New World feline APOBEC3 potently controls inter-genus lentiviral transmission
Source: Retrovirology. 2018 Apr 10;15:31. doi: 10.1186/s12977-018-0414-5 (PMC5894237; doi:10.1186/s12977-018-0414-5)

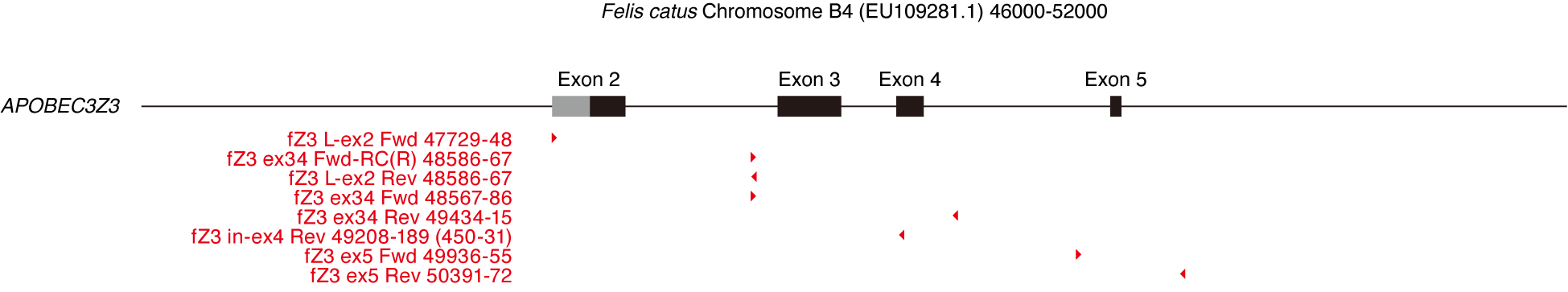

Supplement: Supplementary file 1 — Additional file 1: Figure S1. Scheme of the feline genome encoding APOBEC3Z3 and the position of the primers used in this study. The scheme used for Felis catus chromosome B4, including the exons of feline APOBEC3Z3, is shown. The primers used for PCR/sequencing are shown as red arrowheads, and the names are identical to those in Table S1. [file 12977_2018_414_MOESM1_ESM.tif]

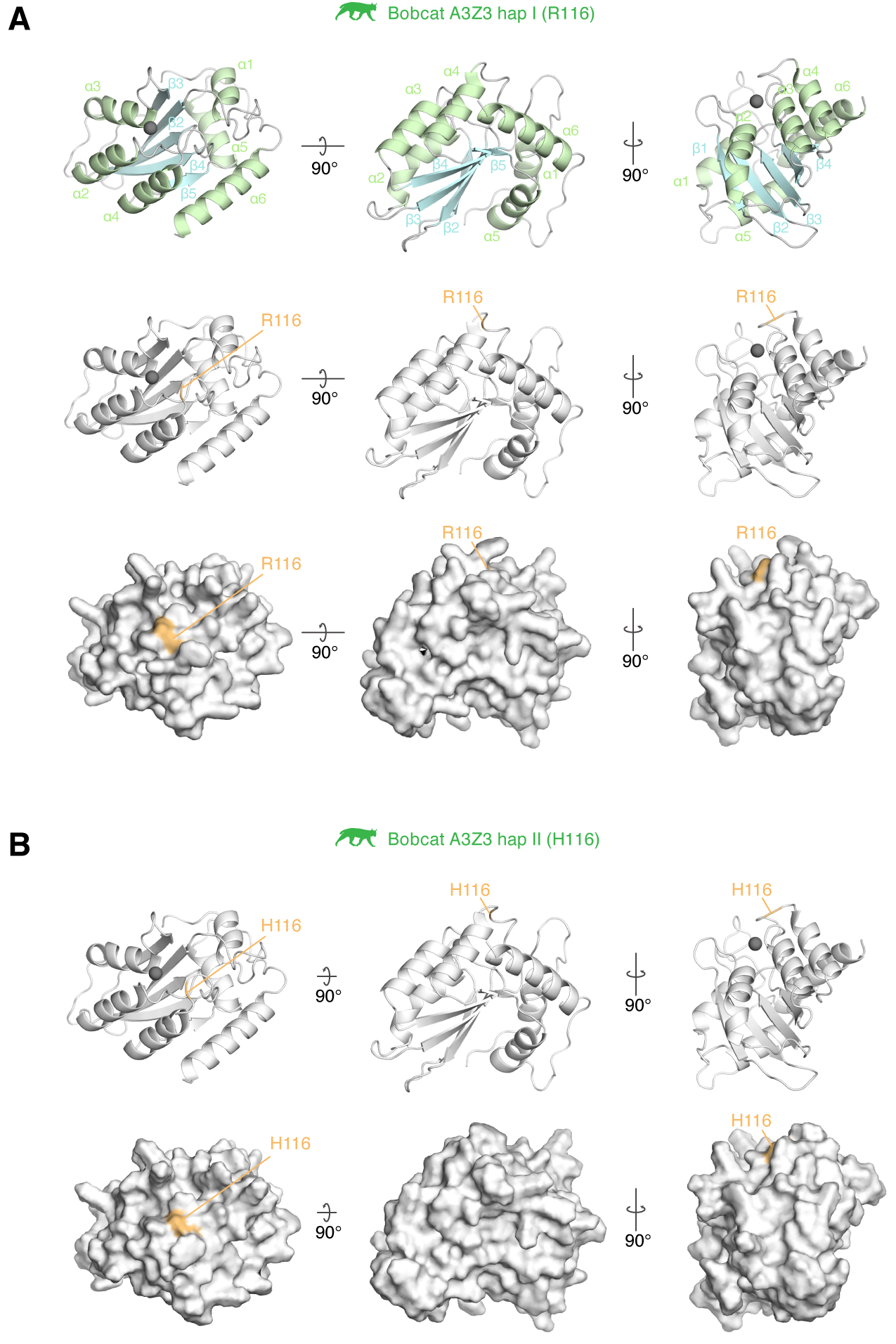

Supplement: Supplementary file 3 — Additional file 3: Figure S2. Structure homology model of bobcat A3Z3 hap II. Cartoon (top and middle) and surface (bottom) models of the A3Z3 protein structures of bobcat hap I (A) and hap II (B) are shown. In the top panel, alpha-helices and beta-sheets are shown in green and pale blue, respectively. Zn2+ is represented as a gray sphere. In the middle and bottom panels, the amino acid that differed between hap I (R116) and hap II (H116) is represented in orange. [file 12977_2018_414_MOESM3_ESM.tiff]
